# Application of optical tweezers in cardiovascular research: More than just a measuring tool

**DOI:** 10.3389/fbioe.2022.947918

**Published:** 2022-09-06

**Authors:** Yi Yang, Zhenhai Fu, Wei Zhu, Huizhu Hu, Jian’an Wang

**Affiliations:** ^1^ Department of Cardiology, Second Affiliated Hospital of Zhejiang University School of Medicine, Hangzhou, China; ^2^ Cardiovascular Key Laboratory of Zhejiang Province, Hangzhou, China; ^3^ Quantum Sensing Center, Zhejiang Lab, Hangzhou, China; ^4^ State Key Laboratory of Modern Optical Instrumentation, College of Optical Science and Engineering, Zhejiang University, Hangzhou, China

**Keywords:** optical tweezer, cardiovascular medicine, heart failure, myosin-actin interaction, cardiomyocytes, microvessels

## Abstract

Recent advances in the field of optical tweezer technology have shown intriguing potential for applications in cardiovascular medicine, bringing this laboratory nanomechanical instrument into the spotlight of translational medicine. This article summarizes cardiovascular system findings generated using optical tweezers, including not only rigorous nanomechanical measurements but also multifunctional manipulation of biologically active molecules such as myosin and actin, of cells such as red blood cells and cardiomyocytes, of subcellular organelles, and of microvessels *in vivo*. The implications of these findings in the diagnosis and treatment of diseases, as well as potential perspectives that could also benefit from this tool, are also discussed.

## Introduction

In the 1970s, Ashkin pioneered the later Nobel Prize-winning tool known as optical tweezers or the optical trap, which exploits the momentum carried by photons to stably suspend microscopic particles and apply forces and torques to them (Killian et al., 2018). This technology went on to make what was once a mere fantasy into a reality: precise and nondestructive measurement and manipulation of molecular biological processes that occur inside the microcosms of cells. This technology found its first application in biosciences in the field of plant research in the 1990s, aiding botanists in the injection of genetic material into plant cells and chloroplasts and in collecting and moving subcellular organelles in rapeseed cells (Greulich and Weber, 1992). Over the past three decades, optical tweezers have proven versatile and widely applicable and have opened doors to revolutionary discoveries in fields such as cancer research, genetic engineering, and immunology (Titushkin and Cho, 2007; Padgett and Bowman, 2011; Greulich, 2017).

**FIGURE 1:**
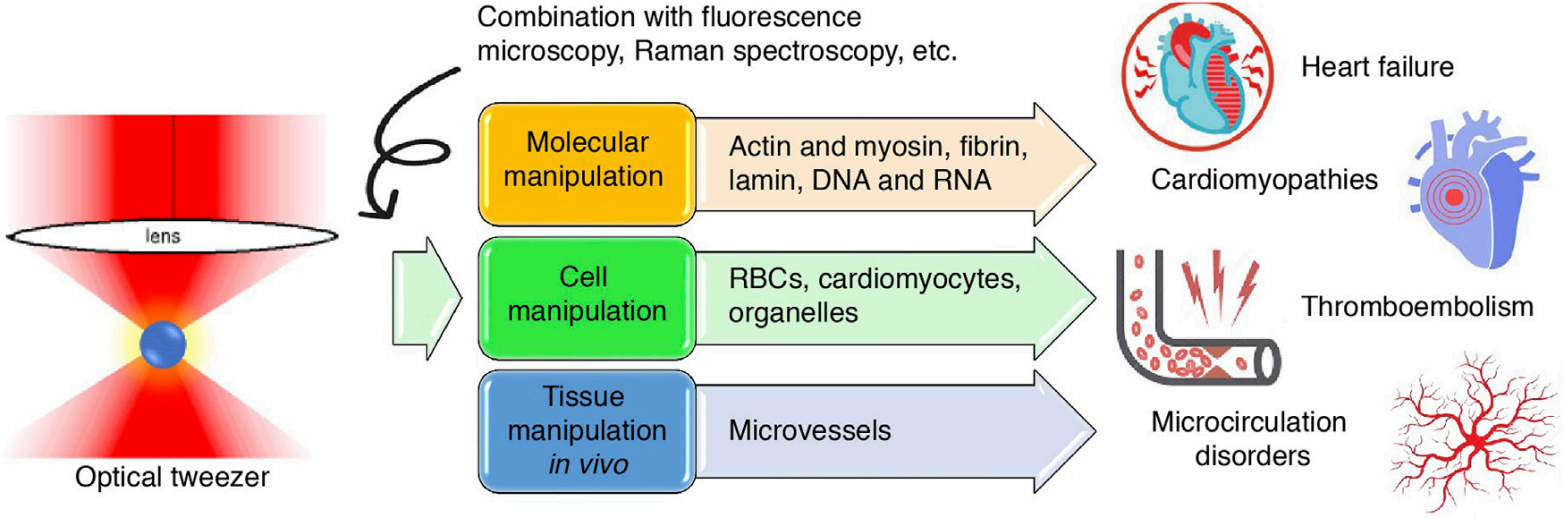
Graphical abstract. Schematic illustration of the application of optical tweezers in cardiovascular research at the molecular, cellular and tissue levels and the inferred value in cardiovascular diseases.

The heart contracts rhythmically to pump blood throughout the circulation. This rhythmic contraction is generated by the coordinated, independent contraction of individual cardiomyocytes, and intracellularly by actin and myosin sliding and pulling relative to one another. As blood is pumped into the circulation, the hemodynamics of both large vessels and capillaries are impacted by a variety of factors, sensitively controlling blood perfusion into tissues and their own internal microenvironments (Barrick and Greenberg, 2021). Understanding the cellular–molecular mechanisms of these processes requires the aid of a sophisticated nanomechanical tool that could measure biomechanical properties such as the force and elasticity of molecules and manipulate them to meet the needs of investigations on a nanoscopic scale. Since the first report of its application in cardiovascular research in the 1990s, optical tweezers have helped cardiovascular scientists not only investigate but also flexibly control the biophysics of single molecules such as proteins (Kopylova et al., 2016; Hwang et al., 2021) and nucleic acids (Wypijewska Del Nogal et al., 2021), cells (Gentemann et al., 2017; Xie and Liu, 2022), organelles (Tang et al., 2007), and microvessels (Liu et al., 2020). These findings, although rooted in the most fundamental of scientific investigations, also have translational importance in putting forth novel drugs in modern medicine. This article reviews the application and findings of optical tweezers in cardiovascular research and the clinical significance of these findings in disease diagnosis and treatment; moreover, we suggest potential fields that could also be studied using this tool (Figure 1).

## Brief glimpse into the optical tweezers

Although photons, the quanta of light, have no mass, they carry momentum. Thus, when light hits an object, force is exerted, which is known as “radiation pressure.” These optical forces are so minute that the mere experimental observation of them in the 1900s was revolutionary (Lebedew, 1901), and no one at that time could have recognized their significance. In the 1960s, the invention of lasers enabled focusing an intense amount of coherent light in a small area (Masuhara, 2021). This inspired Arthur Ashkin, a nuclear physics PhD graduate, to consider the otherwise overlooked practicality of optical forces: theoretically, if light was intense enough and the particle was small enough, the optical force exerted on the particle would be able to accelerate the particle markedly. In 1970, he designed the first optical tweezers, which used two counterpropagating laser beams to accelerate micron-sized neutral particles, described in his groundbreaking work, *Acceleration and Trapping of Particles by Radiation Pressure* (Ashkin, 1970). Early attempts to apply optical tweezers in biological studies were met with undesired outcomes; that is, unlike viruses and bacteria, trapped living cells would explode under the intense heat generated by laser beams. This was resolved by replacing visible argon light with invisible and weakly absorbed infrared light, which caused significantly less heat damage, and in 1987, Ashkin reported damage-free trapping and observation of *Escherichia coli*, yeast cells, human RBCs, and spirogyra organelles (Ashkin et al., 1987).

**FIGURE 2:**
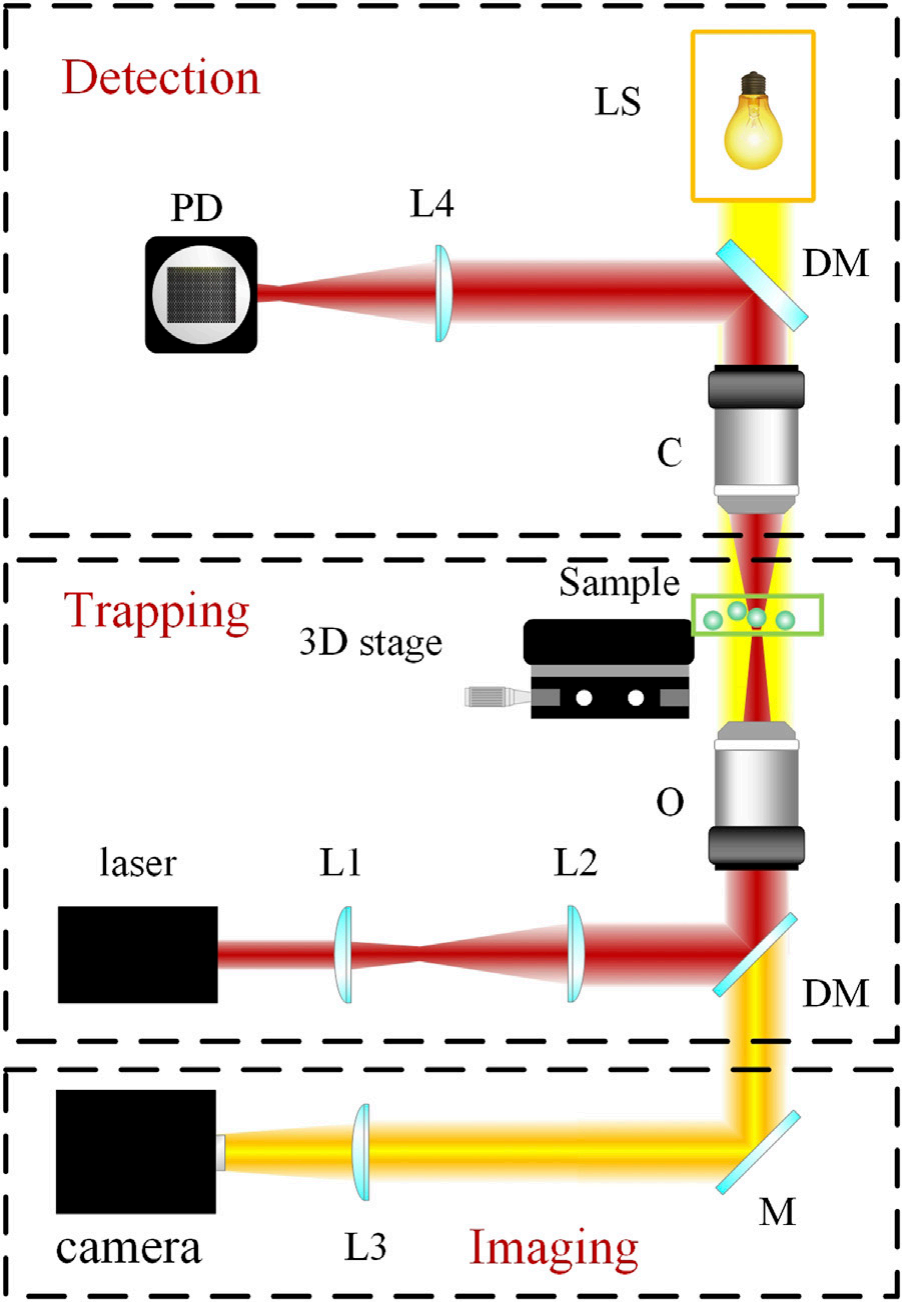
Schematic of setup and principles of a typical optical tweezer. As shown, a typical optical tweezer is composed of three modules: the trapping module, the imaging module, and the detection module. LS, light source; L1, L2, L3, and L4, lenses; DM, dichroic mirror; M, mirror; C, condenser; O, microscope objective; PD, photoelectric detector.

Since the avantgarde works of Ashkin, optical tweezers have not only flourished in a variety of different categories but also morphed from a cutting-edge, unattainable technology to an instrument that is accessible to researchers in many fields (Xin et al., 2020; Gieseler et al., 2021). The three main types of optical tweezers at present include standard optical tweezers, which measure and exert forces and displacements; angular optical tweezers, which measure and exert torques and rotations; and optical tweezers augmented with other instruments, such as fluorescence detection and imaging, as well as Raman spectroscopy, a commonly used optical spectroscopic technique that detects the interaction of light with vibrations from chemical bonds, phonons or other excitations in a material (Raman spectra) to provide a structural fingerprint of the molecules (Bustamante et al., 2021). Although optical tweezers are currently commercially available, many laboratories still build their own instruments. To briefly understand the process of building an optical tweezer from a nonphysicist’s point of view, it requires assembling a commercially available microscope with a suitable laser generator (Greulich, 2017) (Figure 2). The choice of laser depends on the specific range of the spectrum needed in the study. For example, ultraviolet rays have a high absorption coefficient in water and biological materials and are thus used in microablation. In contrast, Nd-doped yttrium aluminum garnet (NdYAG) lasers have a low absorption coefficient in water and biomaterials, and much of their energy transfers momentum; thus, these lasers are often used for trapping (Bustamante et al., 2021). The suspension media may be vacuum, aerosol or liquid; in the case of biomedical studies, liquid optical tweezers are the most often used (Anand et al., 2013; Windey et al., 2019). Simultaneous quantitative trapping of up to hundreds of particles also allows for high-efficiency processing. A detailed protocol for building a customized optical laser apparatus has been elaborately described in previous publications (Jones et al., 2015; Pesce et al., 2015) and will not be the focus of this article.

## Single-molecule studies of cardiovascular diseases

One of the most remarkable attributes of optical tweezers is their rigorous ability to manipulate single molecules, making them an indispensable tool in the study of a variety of molecules that are of significance in cardiovascular diseases (Padgett et al., 2010).

### Studies of myosin and actin and omecamtiv mecarbil

#### Biomechanics of myosin and actin

**FIGURE 3:**
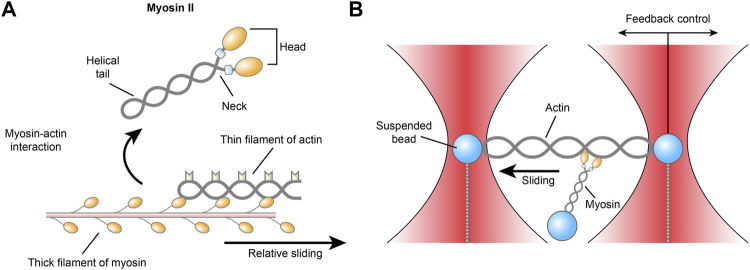
**(A)** Schematic illustration of the actomyosin interaction and **(B)** the three-bead geometry optical tweezer for measuring actomyosin.

Among the molecules of cardiovascular importance studied earliest by optical tweezers are myosin and actin, together known as actomyosin, the laboring proteins that pull together inside single cardiomyocytes to generate myocardial contraction. Myosins are a superfamily of ubiquitously conserved motor proteins distributed in all eukaryotic cells. Different isoforms have specialized functions, but all myosins share the basic function of binding to actin, catalyzing ATP hydrolysis and producing force. Among the 20 isoforms, myosin II, also known as conventional myosin, is the type that is responsible for producing muscle contraction in most animal cells and the only isoform of myosin that polymerizes into filaments. Myosin II has two globular heads with actin-binding ATPase domains. With each power stroke, the heads undergo a conformational change in a two-step manner, while the energy is released from ATP hydrolysis and the myosin climbs one step forward or backward along the actin filament (Veigel et al., 1999; Hwang et al., 2021) (Figure 3). Myosin II consists of heavy chains and light chains that stabilize the neck and modulate motor activity. There are two isoforms of myosin II heavy chains, the α and β isoforms, which have differential ATPase activity and are differentially expressed. Within the heart, the β-isoform is predominantly expressed in the ventricle, while the α-isoform is mainly found in the atrium (Walklate et al., 2021).

Despite rigorous research, it is still unclear how single myosins behave and how they collectively impact cardiac contraction on the whole. As early as 1998, an optical trap with a three-bead geometry was described as an apparatus for measuring the biomechanics of actomyosin. An actin filament is suspended between two polystyrene or ceramic beads held in dual optical traps in adenosine triphosphate-containing (MgATP) medium, while a third bead coated with myosin is placed near the actin to allow the proteins to interact. The actomyosin interaction facilitates the hydrolysis of ATP, which releases energy that is transduced into mechanical work in the form of displacement and force events of the dual beads (Finer et al., 1994). Across more than two decades, although the exact construction of the instrument used by different labs may vary slightly—for example, actin may be attached to one bead instead of two—the core techniques of the optical tweezers used in single-molecule studies have remained the same to this day (Ishii et al., 2018).

For healthy individual myosin II molecules, every ∼100 × 10^−21^ J of energy released from ATP hydrolysis can be converted to a force of ∼10 pN and a displacement of 5–10 nm, adding up to a total of ∼50 × 10^−21^ J of mechanical work (Takagi et al., 2006). This efficiency is altered in mutant myosins isolated from biopsies of patients with hypertrophic cardiomyopathy (HCM) and dilated cardiomyopathy (DCM). However, accumulating evidence has shown that although HCM caused by different mutations exhibits similar clinical manifestations, their myosin functions are altered differently; thus, the early assumption that all HCM mutants are hypercontractile is false. These alterations may include changes in myosin ATPase activity, affinity for actin, and velocity of translocation on actin (Dantzig et al., 2006). R403Q mutation is one of the deadliest mutations, causing 2.3-fold higher ATPase activity, 2.2-fold greater average force generation and 1.6-fold faster actin filament sliding. Using optical tweezers, it was demonstrated that there was no significant difference in the unitary force or displacement generated by wild-type and mutant myosins; thus, the R403Q mutation may have disturbed the mechanical coordination of the two heads (Tyska et al., 2000). Myosin mutants from HCM (R403Q, R453C) and DCM (S532P) were compared using the load-clamp laser trap assay, and it was found that maximal force-generating capacity increased 50% in R403Q-myosin and 80% in R453C-myosin and decreased 65% in S532P-myosin (Debold et al., 2007; Sommese et al., 2013). The R712L mutant myosin from HCM was shown by optical tweezers to have inhibited actin-gliding motility and a working stroke decreased by 4-fold but normal ATPase kinetics and actin-attachment durations (Snoberger et al., 2021).

Apart from quantitative measurements, the direction preferences of the cardiac myosin power stroke have also been addressed using optical tweezers. In a 2021 study, cardiac and fast skeletal myosins were set to pre-, post-, and in-between stroke positions in phosphate solutions, and it was found that unlike fast skeletal myosins, which stayed mainly in the poststroke position, cardiac myosins primarily stayed in the prestroke position. A simulation further confirmed that the reverse stroke is executed more frequently by cardiac myosin, which may be key to characterizing the force output of cardiac muscle (Hwang et al., 2021).

#### Understanding omecamtiv mecarbil

Optical tweezer studies of myosin made translational contributions to the development of a group of cardiac myosin activator drugs as well as an essential understanding of their mechanisms. Traditional anti-heart failure drugs such as β-adrenergic agonists and phosphodiesterase inhibitors are burdened by adverse effects such as proarrhythmia and increased myocardial oxygen demand. Unlike these drugs, which act on the mechanisms upstream of contraction, the cardiac myosin activators act directly on the actomyosin cycle, producing significantly fewer mechanism-related adverse effects on cardiac function. Omecamtiv mecarbil (OM) is the leading drug in this group. OM is currently undergoing phase 3 clinical trials (Teerlink et al., 2020a; Teerlink et al., 2020b). Clinical outcomes to date show that OM boosted left ventricular systolic function, as reflected by increased systolic ejection time, ejection fraction (EF), and improved myocardial strain, while decreasing left ventricular systolic and diastolic volumes, natriuretic peptide concentrations, and heart rate (Biering-Sørensen et al., 2020). Patients with heart failure with reduced EF (HFrEF) who received OM had a significantly lower incidence of heart failure events (37.0%) than those who received placebo (39.1%) (*p* = 0.03) (Teerlink et al., 2021a). This therapeutic benefit was greater as baseline EF decreased (Teerlink et al., 2021b). The favorable effect of OM in right ventricular dysfunction associated with pulmonary hypertension has also been suggested by a clinical trial of a total of 448 patients (Biering-Sørensen et al., 2021). Notably, this ATPase stimulation effect of OM is specific to α- and β-myosin from cardiac and slow skeletal muscles and effective in both healthy and failing human hearts (Nanasi et al., 2018).

Despite positive clinical results, the mechanism of OM on myosin was initially only vaguely understood until an inherent characterization of its molecular actions was achieved using optical tweezers. Although OM is termed by most studies as a myosin activator, the word *activator* actually comes from the dated understanding of OM’s effects in terms of improving cardiac contractile function but does not quite accurately describe the molecular effect of OM on myosin biomechanics, as chronically activated myosin activity would lead to pathological cardiac hypertrophy (Malik et al., 2011). In 2018, a study using optical tweezers demonstrated that OM suppressed the myosin working stroke, prolonging actomyosin attachment and cooperatively activating the thin filament (Woody et al., 2018). OM selectively binds to the S1 domain of the heavy chain of cardiac β-myosin at the site between the converter domain and the relay helix, producing a conformational change in the nucleotide-binding domain, enhancing the ATPase activity and mechanical efficiency of the head and thereby accelerating inorganic phosphate production, which is the rate-limiting step of the actomyosin cycle, producing more force-generating cross-bridges and augmented amplitude and duration of contractions (Nanasi et al., 2018). In summary, OM exerts a multifaceted effect on the complicated actomyosin cycle that cannot be summarized as simply “activating” or “inhibiting.” It was further shown using optical tweezers that therapeutic concentrations of OM produced a more significant benefit on the contractile function of ventricular myosin than on that of atrial myosin (Shchepkin et al., 2020). In particular, the mutation R712L of HCM myosin is adjacent to the OM binding site, and it was found using optical tweezers that although OM inhibited the working stroke of healthy myosin, it rescued the decreased working stroke in the R712L-myosin (Snoberger et al., 2021). These findings underscore the importance of optical tweezers in the development of actomyosin-targeted drugs.

### Studies of other single molecules

Apart from the intricate actomyosin interaction, optical tweezers have been a useful tool in the study of many other molecules in cardiovascular diseases.

#### Fibrin interactions

Thrombosis is the process by which fibrin fibers polymerize into a blood clot ligated by factor XIIIa in an artery or vein. Fibrin polymers are one of the most viscoelastic polymers in nature, and these biomechanical properties of fibrin polymers are particularly crucial to their roles in hemostasis and thromboembolism. During hemostasis, fibrin clots form in response to injury and seal the injured site, minimizing blood loss. Mechanical stretching of the wound may break the clot, directly impacting healing time. Thromboembolism is when a blood clot, usually formed in large deep veins in the leg or in a fibrillating atrium, breaks loose, possibly due to movement, is carried away in the blood flow, and occludes smaller downstream vessels, usually in the lungs or in the brain, causing deadly, paralyzing infarctions (Feller et al., 2022). In the clinical context, the biomechanical properties of thrombi are determining factors for the prevention of thromboembolic diseases and for improving the efficiency of treatments such as endovascular thrombectomy (Boodt et al., 2021; Kwon and Park, 2022). Despite the underlying therapeutic potential, the biomechanics of fibrin polymers have been insufficiently investigated.

In the past two decades, John W. Weisel and others conducted a series of studies on the mechanisms of fibrinogen conversion into fibrin, fibrin polymerization, platelet aggregation at clots, and their interior intermolecular interactions, resolving many mysteries surrounding fibrin activity, and optical tweezers proved to be a useful tool in many of these studies. In 2005, Weisel and others first demonstrated the use of an optical tweezer coupled with a light microscopy to measure the elasticity of individual fibrin fibers in a clot. Blood clots were formed *in vitro* in the presence of factor XIII. Polystyrene latex beads were attached to fibers by perfusion, while an individual bead in the middle of a fiber was identified and trapped by the optical tweezers. Force was applied to the clot in an oscillatory manner, and parameters such as the elastic moduli were calculated from images of the clot (Collet et al., 2005). This initial study provoked curiosity about the interior architecture of clots and the structural basis of their viscoelasticity, which were explored in a series of subsequent works by Weisel. In 2012, Weisel used optical trap-based force spectroscopy to study the molecular mechanism of fibrinogen αC polymer formation in fibrin. It was shown that fibrinogen αC-domains self-associated at their N-termini, while their C-termini interacted with the αC-connectors that harnessed the αC-domains to the bulk of fibrinogen, bolstering the structure of fibrinogen αC polymers and orienting their reactive residues for robust cross-linking by factor XIIIa (Tsurupa et al., 2012). Fibrinogen is converted to fibrin, which has sites such as knobs and holes that facilitate their aggregation, and the response of these bonds under mechanical loads is unknown. In 2018, Weisel again used an optical tweezer-based single molecule forced unbinding experiment to demonstrate that inside fibrin polymers, the strength of inter-fibrin knob-hole noncovalent bonds first increased with force and then decreased after the force exceeded a threshold value (35 pN). This is made possible by the structure of a movable flap near the knob-hole A:a complex with a weak calcium-binding site γ2, which serves as a tension sensor. When force causes the tension sensor flap to dissociate from the B domain and translocate to knob “A,” closure is induced in hole “a,” leading to increased binding affinity. This is significant in that in blood vessels *in vivo*, applying forces just below 35 pN on the A:a knob-hole bonds of fibrin may be favorable for the formation of nascent fibrin clots (Litvinov et al., 2018).

Platelets are an essential participant in pathological thrombosis. Integrin αIIbβ3 binding to fibrinogen and fibrin is the initiation step of platelet aggregation. αIIbβ3 inhibiting agents such as abciximab and tirofiban are widely used to prevent platelet aggregation as adjuncts for percutaneous coronary intervention. Optical tweezers were able to facilitate quantitative biophysical studies of αIIbβ3 interactions. In 2018, Weisel employed an optical tweezer-based system to quantitively compare αIIbβ3 binding to fibrinogen, monomeric fibrin and polymeric fibrin fibers and found that the stability of αIIbβ3 binding to these molecules was in the order of fibrin polymer>fibrin monomer>fibrinogen. αIIbβ3 bound to fibrin polymer in two ways, one with rupture forces of 30–60 pN and another with rupture forces >60 and peaking at 70–80 pN (Höök et al., 2017).

Elevated fibrinogen in blood is also a risk factor for the aggregation of red blood cells (RBCs) in hemorheological diseases. Unlike those of fibrinogen-induced platelet aggregation, the mechanisms of fibrinogen-induced RBC aggregation remain poorly understood. To investigate fibrinogen-induced RBC aggregation, a holographic optical tweezer combined with a fluorescence microscope and microfluidic system was built. Fibrinogen molecules were fluorescently labeled, and the RBCs were trapped in an incubation chamber and constantly flushed with phosphate-buffered saline (PBS) containing fluorescent fibrinogen. The numbers of proaggregant fibrinogen molecules absorbed onto the surface of RBCs were calculated based on the fluorescent signals from the cells, and the decay of the signal reflected fibrinogen desorption. This experiment demonstrated that αIIbβ3 inhibition triggered a marked decrease in fibrinogen adherence to RBCs, relieving RBC aggregation (Semenov et al., 2020). These studies presented a better understanding of fibrin and thrombi, providing laboratory evidence for translation into therapeutic strategies for thromboembolic diseases such as pulmonary embolism and myocardial and cerebral infarction.

#### Lamin mechanics

Lamins are mammalian nucleoskeletal proteins that polymerize into type V intermediate filaments (IFs) and form a meshwork to uphold the structure and rigidity of the nucleus. To do this, tension in the molecule is needed. Mutation of A350P in lamin A principally affects cardiac muscles and leads to dilated cardiomyopathy. In a 2021 study, a pulsed optical tweezer was used to detect the viscosity parameters of wild-type and A350P lamin A, and it was found that the complex viscosity for A350P lamin A was higher than that of wild-type lamin A, which may translate into nuclear plasticity (Mukherjee et al., 2021). Moreover, mutation of the lamin A/C gene D192G also led to weakened tunneling nanotubes (TNTs) and compromised cell–cell adhesion in neonatal rat ventricular fibroblasts, as shown by biomechanical studies using optical tweezers (Lachaize et al., 2021). To date, the topic of lamin mechanics is still vastly unstudied, and this application of optical tweezers could definitely be extended to examine the mechanics of other mutant lamins, as well as any other intermediate filaments.

#### Titins

Titins are gigantic proteins with molecular lengths exceeding 1 µm that act as molecular springs in the passive force of muscles. Titin biophysical properties have fundamental importance in cardiac functions, and mutations in the titin gene (*TTN*) are recognized causes of cardiomyopathies such as dilated cardiomyopathy and hypertrophic cardiomyopathy (Hershberger et al., 2021). The autosomal-dominant hereditary truncating A-band *TTN* variants (TTNtvs), for instance, are the most frequent genetic cause of dilated cardiomyopathy, identified in up to 25% of familial dilated cardiomyopathy cases (Tabish et al., 2017). Genetic testing of individuals with a family history of cardiomyopathies over at least three generations is recommended by international guidelines (Hershberger et al., 2018). Despite the graveness of titin-related cardiomyopathies, the molecular mechanisms of titin and its roles in cardiomyopathies remain complex and not fully understood.

Advances in optical tweezers technology have allowed for mechanistic investigations of the fundamental functional characteristics of titin. Titin molecules mainly comprise 244 tandem foldable protein domains, binding regions, and the elastic PEVK domain (Tskhovrebova et al., 1997; Bianco et al., 2007). Using high-resolution optical tweezers to manipulate and measure their force responses, the tension within titin has been characterized highly nonlinear and entropic. However, when titin is progressively unfolded, force is homogenously distributed along the molecule (Bianco et al., 2015). Titin unfolds at stronger forces (20–30 pN) and refolds under weaker forces (−2.5 pN), while a fraction of the molecule remains consistently unfolded (Kellermayer et al., 1997). The pattern of unfolding of a titin molecule sensitively reflects its previous contractile events, indicating the sophistication of titin in sensing sarcomeric strain (Mártonfalvi et al., 2014). Repetitive stretching induces fatigue in the titin molecule in a physiologically relevant range (0–25 pN); structurally, this is due to nonspecific formation of labile bonds that link random sites of a titin segment (Kellermayer et al., 2001). Further investigations of titin biomechanics may contribute to developing more potent targeted therapies for titin-associated cardiomyopathies.

#### Lipoproteins

Lipoproteins are assemblies that transfer hydrophobic lipids such as fatty acids and cholesterol in blood and other extracellular fluids. Disturbances in the composition and distribution of lipoproteins are implicated in cardiovascular diseases, as certain types of unhealthy lipoproteins infiltrate the arterial wall in the early onset of atherosclerotic lesions (Bornfeldt, 2021). Optical tweezer Raman microspectroscopy has been used to discover signature changes in single lipoprotein particles following consumption of meals, as well as distinct particle distributions caused by different ratios of polyunsaturated to saturated fats in the meals, demonstrating the feasibility of monitoring lipoprotein dynamics at the single-molecule level (Chan et al., 2005).

#### Nucleic acids

Emerging evidence suggests crucial roles for precursor microRNAs (pre-miRNAs) in pathological conditions. These short (∼22 nt), single-stranded, noncoding RNAs hybridize with specific protein-coding messenger RNAs (mRNAs), hindering their translation and regulating gene expression. miRNA-377 regulates angiogenesis and oxidative stress and has been recognized to be markedly increased in the myocardium of failing hearts compared to healthy hearts in humans; the overexpression of miRNA-377 leads to cardiomyocyte apoptosis (Zhu et al., 2016). These reports underscore miRNA-377 as a potential target for treatments and diagnostic biomarkers of heart failure. Optical tweezers were used to pull and unzip single miRNA-377 molecules and study their stability in different ion-containing buffers. It was found that different ions (magnesium, sodium and potassium) demonstrated the capability to stabilize miRNA-377 at different levels by modulating its folding process. Furthermore, the C2 ligand boosted the mechanical stability of miRNA-377, manipulating it or preventing it from folding (Wypijewska Del Nogal et al., 2021). Similarly, using optical tweezers, the study of single nucleotide polymorphisms in DNAs to detect targets as low as 100 pM and associated with coronary heart diseases without the need for amplification was also reported (Koirala et al., 2011). This methodology could easily be adapted to study other nucleic acid sequences to better understand their modulation (Desai et al., 2019).

## Cellular and subcellular studies of cardiovascular diseases

For different cell types with distinct characteristics and functions, optical tweezers have been used to manipulate them in versatile ways for different research purposes (Guck et al., 2010; Wu et al., 2018).

### RBC studies

**FIGURE 4:**
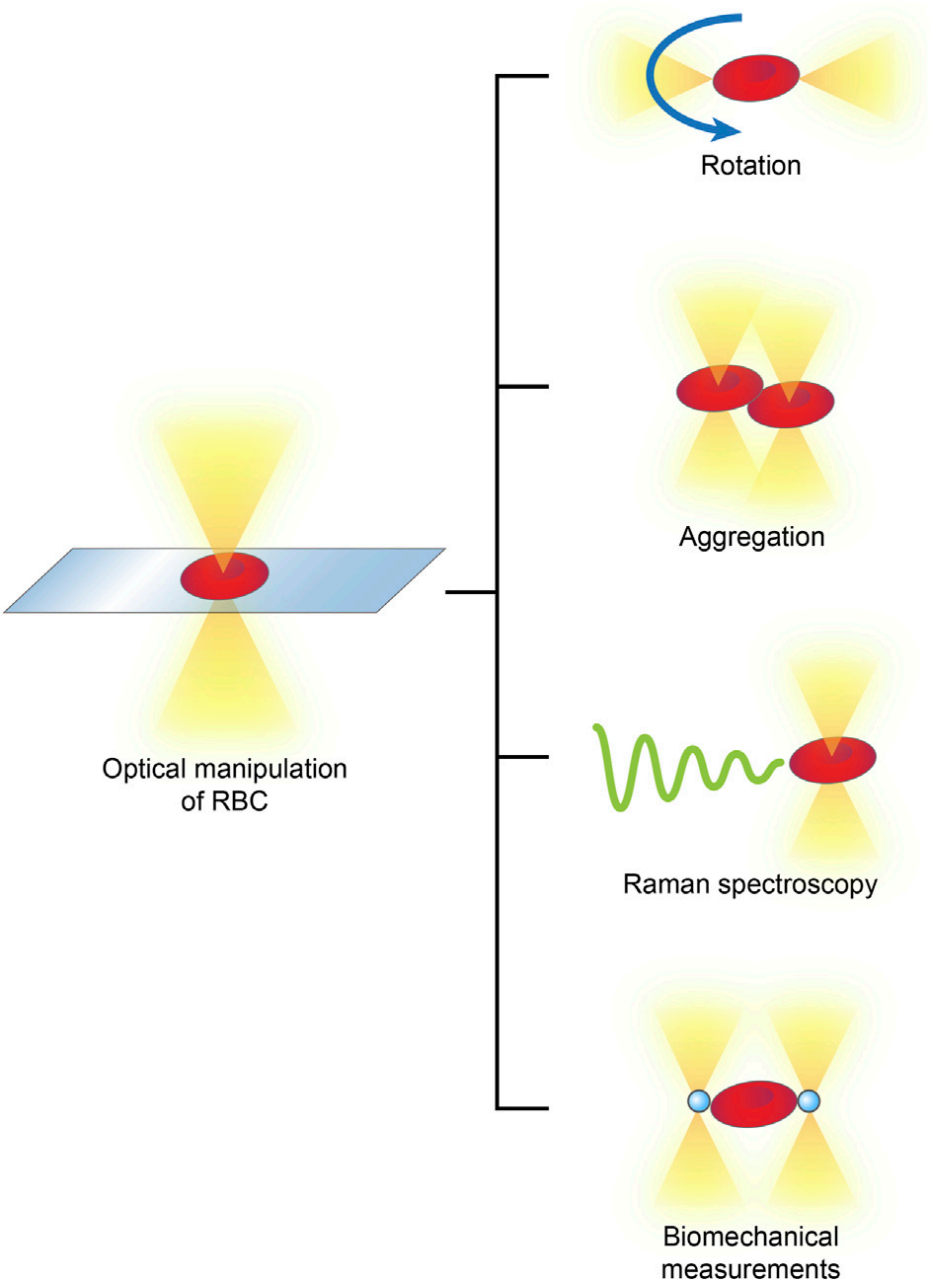
Optical manipulations of RBCs typically include rotation, aggregation, detection of Raman spectra and biomechanical measurements.

RBCs are the major population of peripheral blood cells; they lack nuclei and are homogenous in content. By attaching optically levitated beads to RBC membranes or directly applying forces, an optical tweezer, usually complemented by other techniques, is able to rotate, aggregate and separate RBCs in multifunctional manners and measure their biophysical parameters, such as their membrane elasticity and Raman spectra (Xie and Liu, 2022) (Figure 4).

Diabetes mellitus (DM) is a worldwide threat and the root of cardiovascular complications such as coronary atherosclerosis and diabetic cardiomyopathy. The increased blood glucose-induced alterations in RBC biophysical properties are key to microrheologic complications (Kwon and Song, 2022). The dual optical tweezers technique has been used to study the RBC deformability of DM patients and showed that the RBC deformability index of DM patients was markedly lower than that of healthy controls (Agrawal et al., 2016).

The fact that patients with similar mean glucose levels but vastly different glucose variabilities suffer differential risks of complications raises concern for the management of postprandial hyperglycemia and short-term glucose spikes after meals. In 2021, the effect of short-term hyperglycemic exposure on RBCs was studied using optical tweezers combined with Raman spectroscopy. Integration of optical tweezers with Raman spectroscopy includes the suspension of a material with the optical tweezer and scanning using the Raman spectroscope. Using this technique, RBCs incubated in high-glucose media were compared with control RBCs, and it was found that photoreduction was limited in hyperglycemia-exposed cells, suggesting more permanently oxidized hemoglobin and elevated oxidative stress. Loss of elasticity of the RBC membrane was also detected in hyperglycemia-exposed cells (Singh et al., 2021). In patients with coronary heart diseases (CHDs) and DM, optical tweezers have been employed to observe microrheologic alterations of RBCs. RBCs from healthy people, CHD patients, and patients with both CHD and DM were suspended in the medium of their autological plasma, trapped by tweezers, and placed parallel to each other with a single point of contact under a microscope. The time of spontaneous doublet formation was recorded. It was found that RBCs from CHD patients aggregated faster than those from healthy people, which was further aggravated by the presence of DM (Maslianitsyna et al., 2021). These physical manipulations of RBCs are of clinical significance, as they suggest diagnostic and monitoring tactics for cardiovascular diseases, the methodology of which could be easily extended to studying RBC microrheologics in other pathologies.

### Cardiomyocyte studies

Cardiomyocytes are the predominant myocytes of the chamber walls of the heart that undergo synchronized contraction in response to electric signals from the autorhythmic sinoatrial node. To date, studies on the biophysics of cardiomyocytes using optical tweezers remain preliminary and largely focus on human embryonic stem cell-derived cardiomyocytes (hESC-CMs) and the measurement of their biophysics. One typical study reported measurements of the mechanical stiffness of hESC-CMs using optical tweezers, which showed that stiffness increased after cardiac differentiation, possibly due to increasing myofibrillar structure (Tan et al., 2012). Optical tweezer studies of cardiomyocytes are largely preliminary because once cardiomyocytes mature and become contractile, they themselves contract with forces on the order of several *μ*N, while the forces exerted by an optical tweezer are less than 1 μN to avoid injuring the cells; thus, forces exerted by the optical tweezers are too small to manipulate a contracting cardiomyocyte.

Nevertheless, investigations of cardiomyocytes do not stop at the mere measurement of biophysical properties, and optical tweezers have aided cardiomyocyte studies in other ways. As traditional cell culture techniques lack the intricate arrangement of different cell types that occurs inside the tissue, the laser cell-micropatterning system was developed to arrange cells in a culture dish with high spatial and temporal resolution (Jordan et al., 2005). This technology combines optical trapping, which involves an intensely focused laser beam generated by a high numerical aperture microscope that traps the cell at its focal point, and optical guidance, which involves a weakly focused laser beam generated by a low numerical aperture microscope that traps the cell along its axis and guides it to move in the direction of its propagation. However, this early version of the patterning system is able to pattern only spherical cells and not irregularly shaped cells. By using a computer-mapped spatial light modulator to shape a single microbeam into multiple microbeams, a later version of the micropatterning system is able to place beams around the contour of large and irregularly shaped cells to achieve precise patterning, such as end-to-end tandem alignment of rat cardiomyocytes, without adding surface modifiers (Schmidt et al., 2016). It is thus clear that optical tweezers are not just a nanophysical measuring tool but have versatile potential to be explored in biomedicine. Furthermore, optimization of optical tweezer techniques to strengthen the forces applied to the cell without causing light damage would finally make studying contraction of cardiomyocytes feasible.

### Organelle studies

Mitochondria are ATP-generating cellular fuel engines. In the progression of heart failure, the mitochondria undergo metabolic reprogramming, resulting in less efficient energy production, which causes fatigue and decompensation of the heart. As early as 2007, studies reported the use of optical tweezers for either isolating mitochondria or capturing already isolated mitochondria from rat myocardium to measure their Raman spectra in control and calcium-induced swelling groups (Tang et al., 2007; Reiner et al., 2010). These preliminary attempts paved the way for the later advances using automated optical tweezers to transfer fetal mesenchymal stem cell (MSC) mitochondria to adult MSCs, reported in 2021, resulting in improved antiaging gene expression. The microfluidic, holographic optical tweezer system allowed for the precise and quantitative collection of mitochondria and their transport into recipient cells through endocytosis (Shakoor et al., 2021). Compared with other methods of mitochondrial transplantation reported in the past half decade, such as intramyocardial injection and intracoronary injection, which leave most of the mitochondria functioning in the extracellular space (McCully et al., 2016; Shin et al., 2019), optical tweezer-based mitochondrial transplantation improves the efficiency of mitochondria entering the cell by more than 10-fold, produces far less damage or toxicity, and can holographically transfer up to one to nine mitochondria per cell in just a few minutes (Shakoor et al., 2021).

Currently, although mitochondrial transplantation by intramyocardial injection and intracoronary delivery have been briefly reported to relieve ischemia-reperfusion injury (McCully et al., 2016; Shin et al., 2019), optical tweezer-based mitochondrial transplantation has not been tested in cardiovascular conditions. This prompts us to ask: if heart failure is the irreversible degeneration and loss of cardiomyocytes and mitochondrial energy metabolic switch is the key to this fatigue, could this assumed irreversible progression of heart failure be somewhat reversed using optical tweezer-based mitochondrial transplantation? Furthermore, due to the scarcity of reports, many problems regarding mitochondrial transplantation in myocardial recovery remain unresolved. For example: exactly how do transplanted mitochondria behave after transplantation into a new environment, and what are the cellular and molecular mechanisms? How can we improve transplantation efficiency? Which groups of patients would benefit from this technique (Tang, 2019)? More laboratory, *in vivo*, and even rigorous clinical examinations are needed to answer these questions.

## Manipulation of microvessels *in vivo* in cardiovascular diseases

**FIGURE 5:**
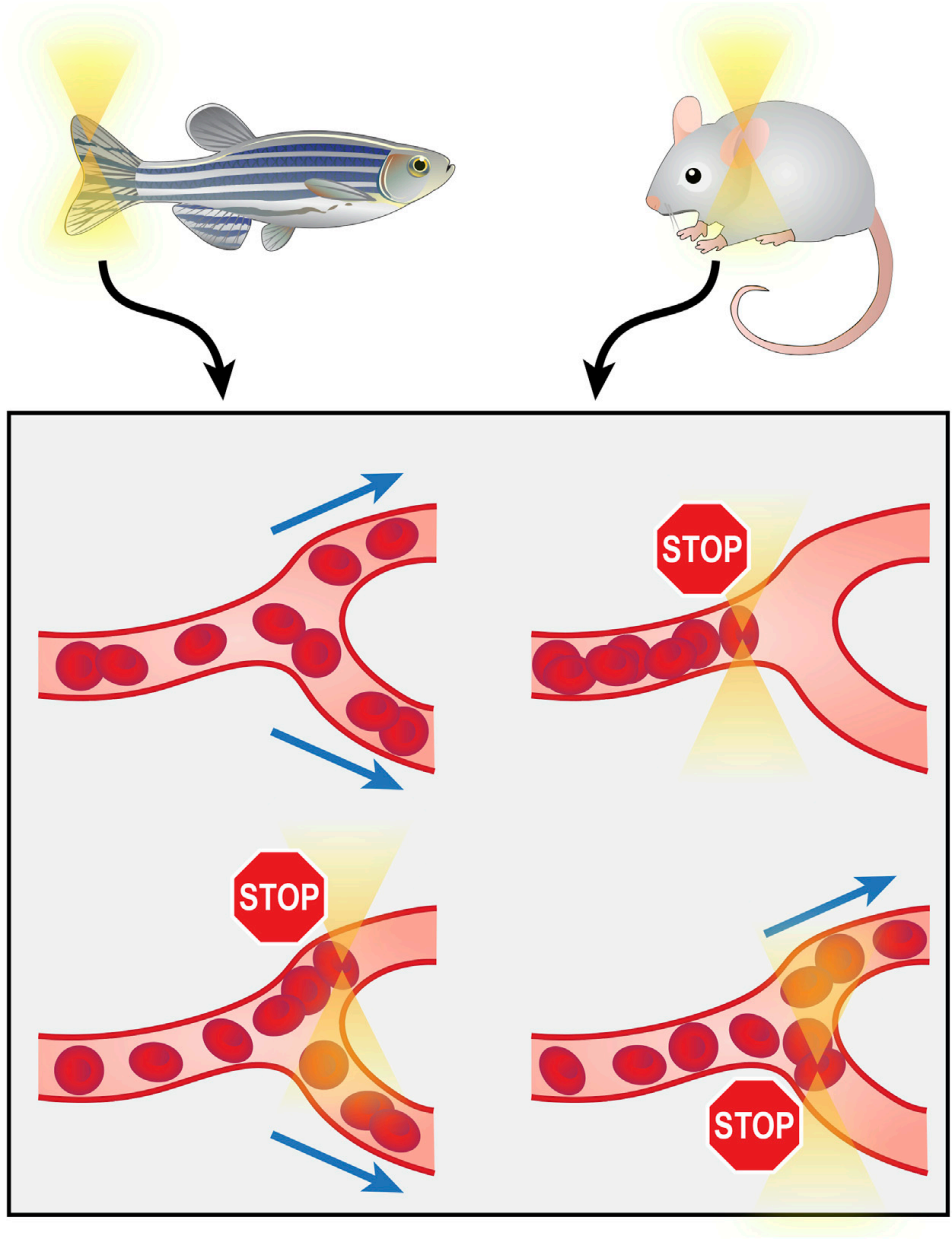
Manipulation of microvessels from zebrafish tail or mouse ear *in vivo* using optical tweezers.

As the aforementioned optical tweezer manipulations all involved the isolation of molecules or cells from their organic environment for *in vitro* operations, the requirement for separation from tissue limits the clinical practicality of these techniques. Directly operating on biological components in their *in vivo* environment would provide more clinical significance. The recently reported (2020) technology of *in vivo* manipulation of microvessels achieves this goal. The optical tweezers were integrated with a microfluidic system on the chip scale, and by trapping and rotating RBCs in capillaries of the zebrafish tail and mouse ear, they demonstrated the capability of targeted switching, directional enrichment, redirecting and rotary actuation of capillary blood flow (Harlepp et al., 2017; Liu et al., 2020) (Figure 5). These investigations of microvessels hold substantial significance, as the importance of microcirculation has gained research attention, and microcirculation dysfunction has been found to be a major cause of a wide range of diseases (Zhang et al., 2019).

Despite the advantages of optical tweezers in *in vivo* manipulations, such as biocompatibility and noninvasiveness, two major obstacles limit their application *in vivo*. One is the depth of operation. The operation depth depends on the depth of the trap generated by the lasers, which is typically less than 100 μm (Liu et al., 2020). Another is that existing reports mostly focus on clear, thin tissues, such as the zebrafish tail and mouse ear, that allow for better transmittance of light to form firmer traps, and no study has been done on thick parenchymal tissues such as the cardiac wall, as they would be difficult to penetrate. Conquering these two problems and achieving deeper and firmer intramuscular trapping *in vivo* could open the door to intracardiac manipulations.

## Discussion and future perspectives

Over the past few decades, the concept of precision and accuracy are becoming more and more emphasized in medical technologies rather than non-specificity and massiveness. Procedures such as percutaneous transcatheter cardiac surgeries and targeted drug deliveries in clinical medicine are showing advantages of producing more effectiveness and less complications. In basic medical research, several methods of single-cell manipulation have been developed which have shown remarkable potential in understanding and investigating cellular processes at the most minuscule level (Shakoor et al., 2022). Widely-used single-cell manipulation techniques include glass microneedles, which are glass capillaries that come in a variety of sizes, robotically or manually controlled, microfluidics, which are controlled microchannel networks that perform cellular and molecular experiments, magnetic tweezers, which perform operations on cells using magnetic field-controlled microrobots, and optical tweezers, which employ forces exerted by light to manipulate cells (Shakoor et al., 2022).

Among these methods, the optical tweezers provide non-invasive, sterile and automated single-cell or single-molecule manipulation with high efficiency and accuracy, and are versatile in application in procedures such as cell manipulation and organelle extraction and transfer. Since its invention, optical tweezers have brought forth rapid developments in a variety of scientific fields. Nevertheless, disadvantages of the optical tweezer, compared with other methods, include higher cost, lower trapping force, and lower throughput. These disadvantages pose critical limitations in further application of the optical tweezer in cardiovascular science and biomedical science in general. One major limitation is its insufficiency to operate *in vivo*. Due to the inability of light to penetrate strongly through thick tissues, current *in vivo* applications of optical tweezers are merely limited to thin and relatively transparent tissues such as zebrafish tail, and no more in-depth *in vivo* application have been reported. Nonetheless, many *in vivo* conditions could prospectively be benefitted from optical tweezer manipulation, if made possible: manipulation and treatment of plaques within the arterial wall, microvessel disorders, *etc*. One potential approach to tackling this would be combining percutaneous interventional catheters with optical tweezers, generating optical tweezers transcatheter to perform *in vivo* procedures. This would, however, expose optical tweezers to challenges of overcoming intravascular hemodynamics and others. One other limitation of optical tweezers is low throughput, making it unable to operate on mass quantities of cells that are sufficient for assays such as mass spectrometry and proteomic analysis. This would require the subsequent assays for the cells to have higher accuracy to detect differences from smaller sample sizes, such as the single-cell sequencing technology.

Besides the reported applications of optical tweezers in cardiovascular studies, many applications of them in other fields of biomedicine have not yet been practiced in cardiovascular studies, but would be potentially worthy of investigation. For example, optical tweezers have been adopted as sensitive transducers in fields such as microrheology (Robertson-Anderson, 2018). Dermal fibroblasts and human fibrosarcoma cells are known to regulate stiffness and other microrheological parameters of their local extracellular matrix. Multi-axes optical tweezers active microrheology have been used to measure multi-dimensional matrix stiffness as affected by collagen concentration and matrix type (Jagiełło et al., 2022). Similarly, cardiac fibroblasts mediate myocardial fibrosis and are impacted by a variety of factors such as pressure and volume overload, paving way to end-stage heart failure. Optical tweezers may be helpful in studying of peri-cardiac fibroblast microrheologics, which have not yet been reported. Microrheologics of other micro-environments, such as blood micro-rheology inside an aneurysm, are other intriguing topics for further investigations.

The past three decades have seen concept-changing research discoveries made by optical tweezers in the field of cardiovascular studies. However, as more knowledge is gained, more unexplored ground becomes recognized. In the near future, we anticipate higher operational efficiency and more versatile manipulation of molecules and cells and improved manipulation in deeper, compact tissues *in vivo* to allow more *in vivo* investigations. As an interdisciplinary topic emerging as a research focus, the application of the optical tweezers in cardiovascular science will surely inspire more multifunctional innovations in the near future, which may both accelerate the emergence of leading-edge biomedical technologies and deliver attainable health care into everyday life.
