# Corrigendum: Application of optical tweezers in cardiovascular research: More than just a measuring tool

**DOI:** 10.3389/fbioe.2022.1075082

**Published:** 2022-11-03

**Authors:** Yi Yang, Zhenhai Fu, Wei Zhu, Huizhu Hu, Jian’an Wang

**Affiliations:** ^1^ Department of Cardiology, Second Affiliated Hospital, School of Medicine, Zhejiang University, Hangzhou, China; ^2^ Cardiovascular Key Laboratory of Zhejiang Province, Hangzhou, China; ^3^ Zhejiang Lab, Quantum Sensing Center, Hangzhou, China; ^4^ State Key Laboratory of Modern Optical Instrumentation, College of Optical Science and Engineering, Zhejiang University, Hangzhou, China

**Keywords:** optical tweezer, cardiovascular medicine, heart failure, myosin-actin interaction, cardiomyocytes, microvessels

In the published article, there was an error in the **Funding** statement. The published version of the **Funding** was, “This work is supported by the National Natural Science Foundation of China (No. 81970230 for WZ), National Natural Science Foundation of China (No. 81870292 for JW), and Major Scientific Research Project of Zhejiang Lab (No. 2019MB0AD01 for HH).” There was one funding source [National Natural Science Foundation for Young Scientists of China (No. 62005248 for Z.F.)] that was omitted. The correct **Funding** statement appears below.

Funding

“This work is supported by the National Natural Science Foundation of China (No. 81970230 for WZ), National Natural Science Foundation of China (No. 81870292 for J’aW), Major Scientific Research Project of Zhejiang Lab (No. 2019MB0AD01 for HH), and National Natural Science Foundation for Young Scientists of China (No. 62005248 for ZF)”.

The authors apologize for this error and state that this does not change the scientific conclusion of the article in any way. The original article has been updated.

